# Urdu Translation and Validation of the Penn Inventory of Scrupulosity-Revised

**DOI:** 10.1192/j.eurpsy.2024.1090

**Published:** 2024-08-27

**Authors:** S. Ghayas, S. Noureen, C. A. Lewis

**Affiliations:** ^1^Psychology, University of Saragoda, Saragoda, Pakistan; ^2^Psychology, Leeds Trinity University, Leeds, United Kingdom

## Abstract

**Introduction:**

Scrupulosity is an Obsessive Compulsive Disorder in which an individual experiences persistent doubts and fears about committing religious and moral sins. Researchers have extensively used the Penn Inventory of Scrupulosity-Revised (PIOS-R), which has been translated into various languages.

**Objectives:**

The present study translated and validated the PIOS-R into Urdu.

**Methods:**

The PIOS-R was translated using the forward-backwards translation method. A sample of 443 Muslim University students (male 224 and female 119) with an age range of 18 to 33 years (*M* = 21.56, *SD* = 2.02) completed the Urdu version of the PIOS-R. Cross-lingual validity was established on a further 60 participants.

**Results:**

Confirmatory factor analysis (CFA) confirmed the two-factor structure of the Urdu version of the PIOS-R. It provided an excellent model fit to the data with chi-square 238.72, CFI = .92, GFI = .93 and RMSEA = .03. The Cronbach’s alpha coefficient of total scale, Fear of God Subscale, and Fear of Sins Subscale was .84, .74, and .78 respectively were satisfactory. The convergent validity of the Urdu version of the PIOS-R was demonstrated with significant positive correlations with measures of anxiety (*r* = .21, *p* <.001) and depression (*r* = .26, *p* < .001).

**Conclusions:**

The Urdu version of the PIOS-R is recommended for use by researchers and practitioners. The results indicated good reliability and validity information for the Urdu version of the PIOS-R, which supports the measure’s utility across cultures and faiths.

**Disclosure of Interest:**

None Declared

